# *Cistanche deserticola*-derived exosome-like nanovesicles target the Microbiota-GABA signaling axis to ameliorate loperamide-induced constipation

**DOI:** 10.3389/fphar.2025.1693366

**Published:** 2025-11-07

**Authors:** Xiaoyu Zhang, Keqiu Yan, Xinkun Bao, Dequn Yang, Xiaoyin Chen, Wenjie Xiao, Jinbo Zhou, Yifan Cai, Hao Wang, Guangjun Sun, Aizhen Lin

**Affiliations:** 1 College of Clinical Medicine of Hubei University of Chinese Medicine, Wuhan, China; 2 Department of Anorectal Medicine, Hubei Provincial Hospital of Traditional Chinese Medicine, Wuhan, China; 3 Hubei Shizhen Laboratory, Wuhan, China

**Keywords:** cistanche deserticola-derived exosome-like nanoparticles, constipation, gut microbiota, γ-aminobutyric acid, intestinal motility

## Abstract

**Introduction:**

Constipation is a prevalent gastrointestinal disorder with limited therapeutic options that often lead to laxative dependence. Gut microbiota and their metabolic regulation represent promising therapeutic targets.

**Methods:**

In this study, we isolated and characterized exosome-like nanovesicles from the traditional Chinese herb *Cistanche deserticola* (CELNs) and evaluated their efficacy in a loperamide-induced mouse model of constipation.

**Results:**

We found that CELNs administration significantly alleviated constipation phenotypes, as evidenced by increased fecal output, water content, and intestinal transit rate, alongside the restoration of colonic histology and goblet cell function. Multiomic analyses revealed that CELNs remodeled the gut microbiota by enriching GABA-producing genera, such as *Lactobacillus* and *Bacteroides*, consequently elevating intestinal GABA levels. This increased GABA specifically activated GABA_A_ receptor subunits α2 and β2/3, thereby enhancing intestinal smooth muscle contraction. Crucially, the therapeutic effects of CELNs were entirely abolished by a GABA_A_ R antagonist, confirming that their action is dependent on GABA_A_ R signaling activation.

**Discussion:**

In summary, our study reveals a novel mechanism through which CELNs enhance intestinal motility via the microbiota–metabolite–host axis, offering a conceptual foundation and a potential nanotherapeutic strategy for the targeted treatment of constipation by modulating microbial homeostasis.

## Graphical Abstract

**Figure:**
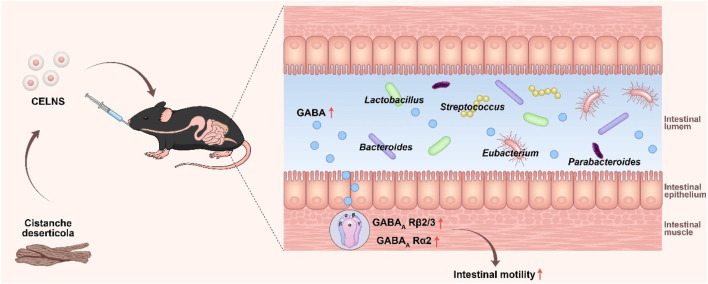

## 1 Introduction

Constipation is gastrointestinal (GI) dysfunction characterized by disorders in intestinal motility, abnormalities in enteric neural function, and delayed colonic transit, leading to difficult defecation. Epidemiological data indicate adult constipation prevalence ranges between 12% and 19%, with higher rates of 30%–40% in the elderly population; additionally, the incidence rate in women is approximately twice that in men (Vlismas et al., 2024; Erhardt et al., 2023). Chronic constipation, which is characterized by persistent and recurrent symptoms, poses significant clinical challenges. Although conventional laxatives provide short-term relief, their prolonged use often results in enteric nerve damage, drug dependency, and symptom recurrence in 30%–50% of patients, underscoring the limitations of current therapeutic strategies (Barbara et al., 2023; Luo et al., 2025). Recent large-scale evidence indicates that constipation is associated with an increased risk of all-cause mortality, with pooled hazard ratios of 1.10 (95% CI 1.02–1.19) in the general population, 1.40 (95% CI 1.16–1.68) in patients with chronic kidney disease, and 1.85 (95% CI 1.28–2.67) in those with heart failure, highlighting its role as a significant marker of systemic health burden, particularly in high-risk subgroups (Liu B. et al., 2025). This positions constipation not merely as a localized gastrointestinal ailment but as a potential sentinel marker of systemic health, warranting further investigation into its underlying pathophysiological mechanisms.

Growing evidence suggests that gut dysbiosis, characterized by alterations in microbial composition, specifically reductions in beneficial bacterial taxa and expansions in potentially pathogenic microorganisms, are implicated in the pathogenesis of constipation (Rau et al., 2024). The gut microbiota and its metabolites have emerged as promising therapeutic targets for accommodating intestinal motility, enhancing mucosal barrier integrity, and regulating neural signaling (Hou et al., 2025; Zhang et al., 2025). Of particular interest is the microbial metabolite γ-aminobutyric acid (GABA), which alleviates constipation by suppressing enteric neuronal hyperexcitability and restoring intestinal peristaltic rhythms (Gajic Bojic et al., 2025). Interventions such as GABA-producing probiotic administration or metabolic pathway modulation have demonstrated efficacy in improving gut motility (Xia et al., 2024).

Recently, there has been a focus on plant-derived exosome-like nanoparticles because of their ability to target microbial homeostasis. As natural nanocarriers and intercellular messengers, plant-derived exosome-like nanoparticles traverse the upper GI tract intact under the protection of lipid bilayers, delivering bioactive components to the gut microbiota to regulate its composition or function. For example, broccoli-derived exosomes alleviate loperamide (LOP)-induced constipation in mice by restoring the gut microbiota structure and modulating microbial metabolite profiles, particularly short-chain fatty acids (SCFAs) (Duan et al., 2023). Similarly, garlic-derived exosome-like nanoparticles increase the abundance of *Lactobacillus* and *Lactobacillus reuteri reuteri* while also increasing the levels of anti-inflammatory metabolites such as indole-3-propionic acid, thereby reducing mucosal inflammation and maintaining intestinal homeostasis (Liu X. Y. et al., 2025). These implications underline the potential of plant-derived exosomes as candidates for ameliorating constipation-associated gut dysbiosis.

*Cistanche deserticola*, a traditional Chinese medicinal herb, contains diverse bioactive compounds, including phenylethanoid glycosides (e.g., echinacoside and acteoside) and polysaccharides. Studies have highlighted the anti-inflammatory, antioxidant, immunomodulatory, and prokinetic properties of this herb (Qiao et al., 2025; Yin et al., 2024). Notably, *C. deserticola* polysaccharides modify the intestinal microbial profile, optimize the gut environment, and ameliorate colonic motility disorders (Liu et al., 2022). However, the effects of *C. deserticola*-derived exosomes on constipation remain underexplored.

In this study, we isolated *C. deserticola* exosome-like nanovesicles (CELNs) via ultracentrifugation and evaluated their anti-constipation efficacy in a murine model of LOP-induced constipation. Multiomics analyses, including host colonic transcriptomics, 16S microbiome profiling, and fecal metabolomics, were performed to unravel the mechanisms by which CELNs restore gut health through microbiota and functional pathway regulation.

## 2 Materials and methods

### 2.1 Isolation and purification of the CELNs

*C. deserticola* was provided by the Hubei Provincial Hospital of Traditional Chinese Medicine and identified as a dried fleshy stem by Associate Professor Huanbo Cheng from the School of Pharmacy, Hubei University of Chinese Medicine. Approximately 50 g of dried *C. deserticola* was homogenized in 150 mL of phosphate-buffered saline (PBS) and incubated at 4 °C for 24 h. The fresh liquid was subsequently centrifuged at 4 °C at 1,000 × g for 10 min, 3,000 × g for 30 min, and 10,000 × g for 30 min to remove large particles and fibers. The final supernatant was filtered through a 0.45-μm microporous filter membrane and centrifuged at 4 °C and 100,000 × g for 1 h. The obtained crude extracted precipitate was resuspended in 20 mL of PBS and recentrifuged under identical ultracentrifugation conditions. Finally, the purified CELNs were resuspended in sterile PBS and stored at −80 °C for future use.

### 2.2 Characterization and identification of the CELNs

A 20-μL aliquot of CELNs was deposited onto a copper mesh grid and permitted to precipitate for 1 min. Excess liquid was subsequently removed by absorption with filter paper. The grid was then stained with 20 μL of uranyl acetate for 1 min, followed again by removal of excess solution using filter paper. Samples were air-dried at room temperature prior to morphological and structural analysis by transmission electron microscopy (TEM; HT-7700, Hitachi, JEOL Ltd., Japan).

The obtained CELNs were resuspended and mixed in 1 mL of PBS. Subsequently, 0.2 mL of the suspension was transferred to a cuvette. Particle size distribution and zeta potential were characterized using a Zetasizer Nano ZS analyzer (Malvern Panalytical Ltd., united Kingdom) and analyzed with Origin 2021 software. Separately, CELN concentration and size distribution were determined by nanoparticle tracking analysis (NTA) using a NanoSight LM20 instrument (Malvern Panalytical Ltd., Amesbury, united Kingdom). For NTA, CELNs were diluted in PBS and 10 μL aliquots were analyzed at room temperature.

### 2.3 Cellular internalization

The human colorectal adenocarcinoma cell line HT-29 (Procell Life Science & Technology Co., Ltd., Wuhan, China) was maintained in DMEM containing 10% fetal bovine serum (FBS) and 1% penicillin/streptomycin at 37 °C under 5% CO_2_.

CELNs were labeled by incubation with the lipophilic fluorescent dye PKH26 (5 min, room temperature). Unbound dye was removed via centrifugation (100,000 × g, 4 °C, 1 h), and the pellet was resuspended in 200 μL PBS. PKH26-labeled CELNs were then co-incubated with HT-29 cells (37 °C, 24 h). Following incubation, cells were stained with the nuclear counterstain DAPI and visualized by confocal microscopy.

### 2.4 Animal experiments

Male C57BL/6J mice (6–8 weeks, 20 ± 2 g) were obtained from the Hubei Provincial Center for Disease Control and Prevention (Wuhan, China). Animals were maintained under specific-pathogen-free conditions (12-h light/dark cycle) with *ad libitum* access to food and water. Following 1 week of acclimatization, mice were randomized into six experimental groups (n = 10) as described previously: Control, LOP (loperamide), CELN-L (low-dose CELNs), CELN-M (medium-dose CELNs), CELN-H (high-dose CELNs), and LAC (lactulose) (Zhang et al., 2021a). Twice-daily oral gavage of loperamide (10 mg/kg in 0.2 mL) was administered to all experimental groups, excluding the Controls, for two consecutive weeks. Control animals received equivalent volumes of 0.9% saline via identical administration. Starting on day 7, the CELN group received once-daily gavage of CELNs at gradient doses (25, 50, or 100 mg/kg); the LAC group received lactulose (2.5 g/kg), both in a 0.2 mL volume, for 1 week.

To investigate deeper mechanistic pathways, male C57BL/6J mice were randomized into five cohorts (n = 10 per group): Control, LOP, CELN-H, BIC (bicuculline), and CELN-H + BIC. With the exception of the Control group, all cohorts underwent twice-daily oral gavage with LOP (10 mg/kg) for 2 weeks. Control animals were administered equivalent volumes of 0.9% saline using identical methodology. Beginning on the 7th day, the mice in the CELN-H group were administered 100 mg/kg CELNs via gavage, those in the BIC group were intraperitoneally injected with bicuculline (1 mg/kg in 0.2 mL), and those in the CELN-H + BIC group received 100 mg/kg CELNs via gavage followed by 1 mg/kg bicuculline via intraperitoneal injection 1 hour later. After the experiment, all the mice were euthanized, and serum, colon tissues, and cecal contents were collected and stored at −80 °C for further analysis.

The animal experiments were performed according to the Ethics Committee of the Center for Animal Experiments of Hubei University of Chinese Medicine (HUCMS-42360218) and the National Act on Use of Experimental Animals (China).

### 2.5 Determination of constipation indices

Mouse body weights were recorded daily throughout the experimental period. Following treatment completion, fecal pellets were collected from individual mice and quantified. Fresh stool weight was measured before drying at 60 °C for 12 h to determine dry mass. Fecal water content was calculated as: [(wet weight - dry weight)/wet weight] × 100%.

At the end of the treatment period, mice from each group (n = 10) were randomly divided into two subsets for intestinal transit assessments. One subset (n = 5) was used to evaluate the time to first black stool: after a 12-h fast (following the final drug administration), mice were gavaged with 0.2 mL Indian ink and the time until the first black stool excretion was recorded. The other subset (n = 5) was used to determine the intestinal transit rate: after an identical 12-h fast, mice were gavaged with 0.2 mL of a charcoal meal and euthanized 30 min later to measure the propulsion distance of the charcoal meal in the small intestine.

### 2.6 Enzyme-linked immunosorbent assay (ELISA)

Serum concentrations of motilin (MTL), substance P (SP), and 5-hydroxytryptamine (5-HT) were quantified using commercial ELISA kits (Ruixin Biological Technology Co., Ltd., Quanzhou, China) following manufacturer protocols.

### 2.7 Histopathological staining

Colon tissues were excised and immersion-fixed in 4% paraformaldehyde. Following dehydration and clearing, samples were paraffin-embedded and sectioned at 5 μm. Sections were deparaffinized, rehydrated, and sequentially stained with hematoxylin-eosin (H&E) and alcian blue. After staining, slides were mounted with neutral balsam and air-dried. The mucin-positive area (stained blue), expressed as a percentage of the total mucosal area, was determined using ImageJ (NIH, United States).

### 2.8 Western blot analysis

Proteins were extracted from colon tissues and quantified. Equal protein aliquots were resolved by SDS-PAGE (10% gel) and electroblotted onto PVDF membranes. Membranes were blocked with 5% non-fat milk in TBST (1 h, room temperature), then incubated overnight at 4 °C with the following primary antibodies: anti-glutamic acid decarboxylase 65/67 (GAD 65/67) (sc-365180, Santa Cruz Biotechnology, CA, United States), anti- GABA_A_ receptor subunits β2/3 (GABA_A_ R β2/3) (AB5592, Millipore Corporation, MA, United States), anti- GABA_A_ receptor subunits α2 (GABA_A_ R α2) (sc-7355, Santa Cruz Biotechnology, CA, United States), and anti-GAPDH.

### 2.9 Colon transcriptomic sequencing

Total RNA was extracted from colon tissues using an RNA simple Total RNA Kit (TIANGEN) followed by DNase I (TaKaRa) treatment for genomic DNA removal. RNA integrity was verified using an Agilent 2100 Bioanalyzer (Agilent Technologies, Santa Clara, CA), and concentrations were determined with a NanoDrop ND-2000 spectrophotometer (Thermo Fisher Scientific, Wilmington, DE). The RNA-seq transcriptome analysis was performed by Fujian Manxiu Biotechnology Co., Ltd. Briefly, RNA-seq libraries were prepared from 1 μg of total RNA and sequenced on an Illumina HiSeq xten/NovaSeq 6000 platform (paired-end) after TBS380 quantification. Raw sequencing reads underwent quality assessment via FastQC (v0.12.1) and adapter trimming using Trimmomatic (v0.39). High-quality reads were aligned to the reference genome using HISAT2 (v2.2.1) with default splice-aware parameters. Transcript assemblies were subsequently generated with StringTie (v2.2.1) and quantified as FPKM values.

### 2.10 Amino acid-targeted metabolomic analysis

Amino acid-targeted metabolomic profiling was conducted via liquid chromatography-tandem mass spectrometry (LC-MS/MS). Briefly, colon content samples were homogenized in ice-cold 80% methanol (v/v, containing isotope-labeled internal standards) using a TissueLyser II (Qiagen) at 30 Hz for 3 min. Following centrifugation (12,000 × g, 10 min, 4 °C), supernatants were separated on an ACQUITY UPLC BEH C18 column maintained at 40 °C. Chromatography employed a 0.4 mL/min gradient of solvent A (0.1% formic acid in water) and B (0.1% formic acid in acetonitrile) over 12 min. MS detection utilized a Xevo TQ-S triple quadrupole mass spectrometer in multiple reaction monitoring (MRM) mode with polarity switching. Metabolite quantification was performed by normalizing peak areas to internal standards with calibration curves (*R* ^2^ > 0.99) generated from serially diluted authentic standards. Principal component analysis (PCA) was used to conduct an unsupervised cluster analysis of the amino acid metabolites detected to evaluate the overall differences among samples.

### 2.11 16S rDNA sequencing and analysis

Gut microbiota analysis was performed following established protocols (Zhang et al., 2021a; Zhang et al., 2021b). Briefly, cecal contents were aseptically collected post-euthanasia (n = 5 biological replicates). Microbial DNA was extracted using the FastDNA Spin Kit for Feces (MP Biomedicals, Irvine, CA) with bead-beating homogenization (6.0 m/s for 60 s). The hypervariable V3-V4 region of the bacterial 16S rDNA gene was amplified by PCR with primers 338F (5′-ACT​CCT​ACG​GGA​GGC​AGC​AG-3′) and 806R (5′-GGACTACHVGGGTWTCTAAT-3′). Amplicons were size-selected via 2% agarose gel electrophoresis, purified (QIAquick Gel Extraction Kit, Qiagen), and quantified (Qubit dsDNA HS Assay Kit, Thermo Fisher). Libraries were sequenced on an Illumina MiSeq platform (2 × 300 bp paired-end; MiSeq Reagent Kit v3). Raw sequences were processed in QIIME2 using DADA2 for quality filtering, denoising, and generation of amplicon sequence variants (ASVs). Taxonomy was assigned against the SILVA 138.1 database at a 97% sequence similarity threshold. Alpha diversity was assessed using Faith’s Phylogenetic Diversity (PD) whole tree metric, and beta diversity was evaluated via non-metric multidimensional scaling (NMDS) based on Bray-Curtis distances.

### 2.12 Statistical analysis

Data from biochemical and physiological experiments are presented as the mean ± SEM. Statistical analyses were performed using GraphPad Prism v9.0 (GraphPad Software, San Diego, CA). Intergroup comparisons employed unpaired two-tailed Student's *t*-tests. For multivariate analyses involving ≥2 factors, two-way ANOVA with Šidák’s multiple comparisons correction was applied. Statistical significance was defined as *P* < 0.05. The OmicShare platform was used to create clustering heatmaps and network heatmap.

For RNA-seq (transcriptomic) data analysis, Raw read counts were normalized via Variance Stabilizing Transformation (VST) using the DESeq2 package (v1.40.0) to account for sequencing depth variability. Differentially expressed genes (DEGs) were identified with DESeq2, using thresholds of |log_2_ (fold change)| ≥ 1 and adjusted P-value (padj) < 0.05. DEGs were annotated against the Gene Ontology (GO) and Kyoto Encyclopedia of Genes and Genomes (KEGG) databases using the clusterProfiler package (v4.8.1), with statistical significance determined by hypergeometric tests (padj <0.05).

For 16S rDNA gene sequencing data analysis, PD whole tree was calculated in QIIME2 using the diversity plugin; intergroup comparisons were performed via Kruskal–Wallis test (followed by Dunn’s *post hoc* test) in R’s vegan package (v2.6-4). Bray-Curtis dissimilarity matrices were generated in QIIME2; non-metric multidimensional scaling (NMDS) was visualized in R’s vegan package, with statistical group separation confirmed by ANOSIM (R = 0.72, P < 0.001). Relative abundance differences of genera were tested using ANOVA (for normally distributed data) or DESeq2 (for sparse count data) in R’s phyloseq package (v1.44.0), with padj <0.05 as the significance cutoff.

## 3 Results

### 3.1 Isolation and characterization of the CELNs

**FIGURE 1:**
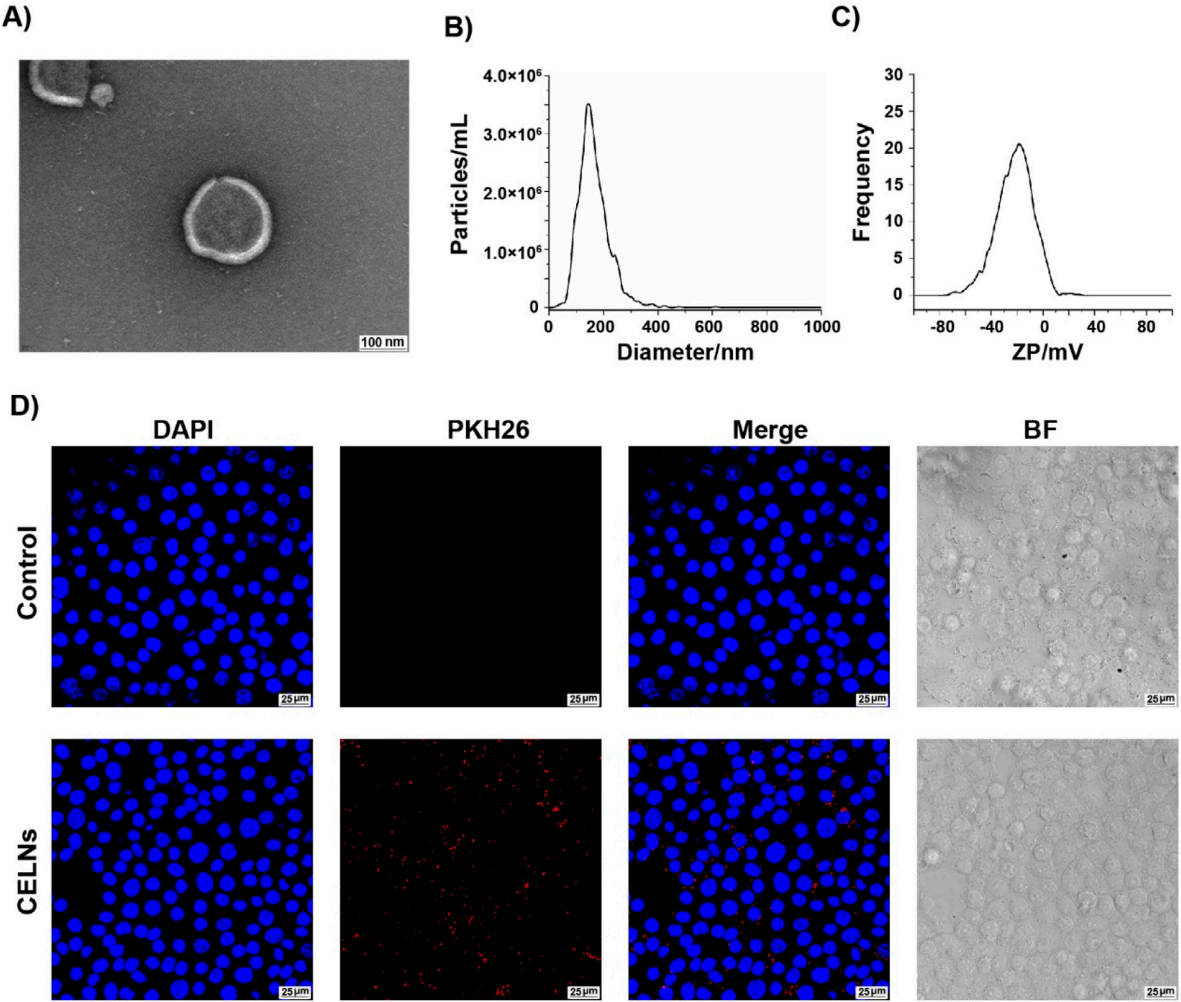
Characterization and cellular internalization of (C) *deserticola* exosome-like nanovesicles (CELNs). **(A)** Transmission electron microscopy (TEM) images of CELNs (scale bar = 100 nm). **(B)** The size of the CELNs was analyzed by nanoparticle tracking analysis (NTA). **(C)** The zeta potential of the CELNs was measured with a ZetaView system. **(D)** PKH26-labeled CELNs (red) were taken up by HT-29 cells. Nuclei are stained with DAPI (blue). Scale bar = 25 μm.

CELNs were isolated from *C. deserticola* via differential centrifugation followed by ultracentrifugation. TEM analysis revealed circular-to-elliptical vesicles with characteristic bilayered membranes exhibiting a mean diameter of 152.6 nm and particle concentration of 4.6 × 10^10^ particles/mL (Figures 1A,B). These morphological and quantitative metrics conform to established exosome-like nanoparticle parameters. Zeta potential measurements confirmed a surface charge of −21.67 mV (Figure 1C). Next, we investigated the biological function of CELNs *in vitro* to determine whether they can be effectively internalized by cells. Red fluorescent CELNs were observed in the cell cytoplasm, whereas no signal was detected in the Control group (Figure 1D), demonstrating that CELNs can be efficiently internalized by HT-29 cells. And based on the UPLC fingerprint, the main active metabolite in CELNs were determined to be echinacoside (Supplementary Figure S1).

### 3.2 CELNs alleviate LOP-induced constipation in mice

**FIGURE 2:**
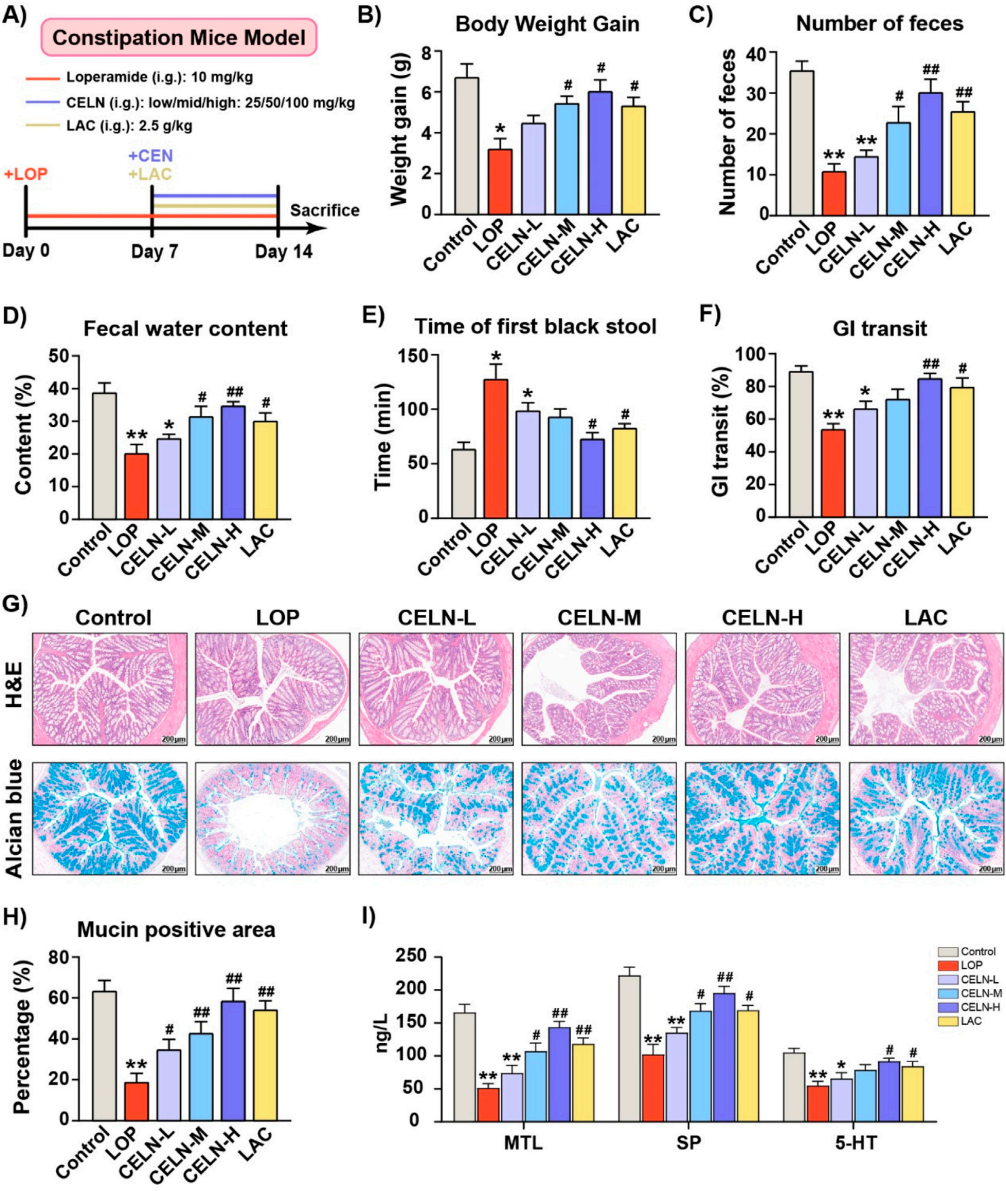
Effect of CELNs on loperamide-induced constipation in mice. **(A)** Schematic of the animal experimental design. **(B)** Body weight gain. **(C)** Number of fecal pellets. **(D)** Fecal water content. **(E)** Time to first black feces. **(F)** Gastrointestinal transit rate. **(G)** Representative images of hematoxylin and eosin **(H,E)**- and alcian blue-stained colon tissues from each group (scale bar = 200 µm). **(H)** The percentage of mucin-positive area (blue staining). **(I)** Serum concentrations of motilin (MTL), substance P (SP) and 5-hydroxytryptamine (5-HT) among the experimental groups. The data are presented as the means ± SEMs. ^*^ *P* < 0.05, ^**^ *P* < 0.01 vs. the Control group; ^#^ *P* < 0.05, ^##^ *P* < 0.01 vs. the LOP group.

To determine the effect of CELNs on constipation, we constructed a mouse model of constipation induced by LOP and administered gradient doses of CELNs, while mice in the Control group received lactulose, which is a known anticonstipation agent (Figure 2A). CELNs treatment clearly suppressed the decrease in body weight in the constipated mice (Figure 2B). The number of fecal pellets and fecal water content in constipated mice were distinctly lower than those in conventional mice, but this difference was reversed by CELNs treatment (Figures 2C,D). Additionally, the mice in the LOP group showed longer times to first black stool defecation and significantly slower GI transit times, and high doses of both CELNs and lactulose markedly improved these durations (Figures 2E,F). Histomorphological analysis of H&E-stained sections revealed constipation-induced colonic mucosal damage, characterized by villous blunting, crypt architectural distortion, and muscularis propria atrophy. CELNs administration restored colonic architecture, demonstrating significant increases in muscularis thickness in the colon (Figure 2G).

Alcian blue staining demonstrated that CELNs treatment significantly ameliorated constipation-induced deficits in colonic mucin production-critical to mucosal barrier function-with mucin levels restored to near-normal baselines (Figures 2G,H). Consistent with the histochemical observations, transcriptomic analysis revealed a marked induction of mucin1 expression in the CELN-H group (Supplementary Figure S2). Hormones and neurotransmitters related to GI motility, such as MTL, and the neuropeptide/neurotransmitter SP, play important roles in promoting GI motility. Furthermore, 5-HT, which functions as both a neurotransmitter in the ENS and a paracrine agent released from enterochromaffin cells, is a critical regulator of intestinal contractions and peristalsis. Constipated mice exhibited significant reductions in MTL, SP, and 5-HT relative to controls. However, the high dose of CELNs significantly increased the levels of these factors, with an effect superior to that of lactulose (Figure 2I).

### 3.3 Systemic regulation of colonic mRNA expression by CELNs in constipated mice

**FIGURE 3:**
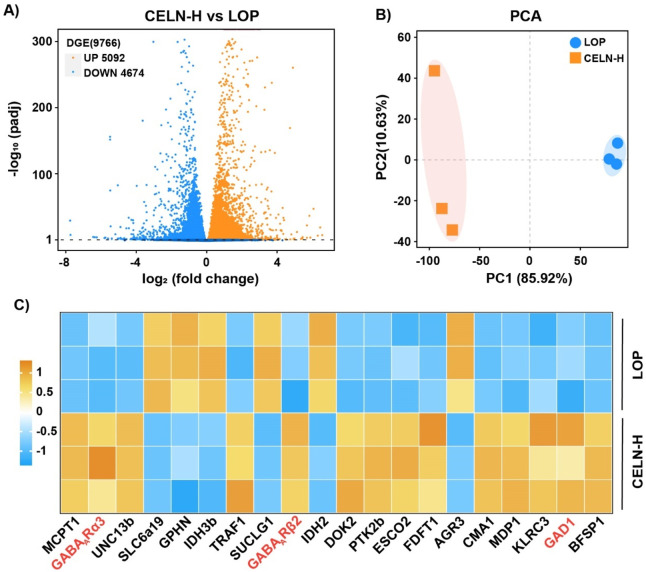
RNA-seq analysis of mouse colon samples. **(A)** Volcano plot showing upregulated (orange dots) and downregulated (blue dots) mRNAs in the comparison. **(B)** Principal component analysis (PCA) plot displaying two distinct clusters that reveal differences in mRNA expression between the two experimental groups. **(C)** Heatmap showing the upregulation (orange) and downregulation (blue) of specific targets in the comparison.

To elucidate the laxative mechanism of CELNs, we performed RNA sequencing to profile transcriptomic alterations in colonic tissues from constipated mice following therapeutic intervention. After normalization and filtering of the RNA-seq data, several differentially expressed mRNAs were identified, 5,092 of which were upregulated and 4,674 of which were downregulated (Figure 3A). Principal component analysis (PCA) delineated three discrete transcriptional clusters, revealing pronounced mRNA expression disparities between experimental cohorts (Figure 3B). Applying thresholds of FPKM >1 and |log_2_FC| ≥1 (where log_2_FC > 1 denotes upregulation and log_2_FC < -1 downregulation), we identified 20 differentially expressed mRNAs with established disease associations. Notably, the CELN-H group exhibited significantly elevated expression of GABA_A_ R subunits (including α3 and β2) and GAD1 compared to LOP controls (Figure 3C).

### 3.4 The effects of the CELNs depend on GABA_A_ R pathway activation

**FIGURE 4:**
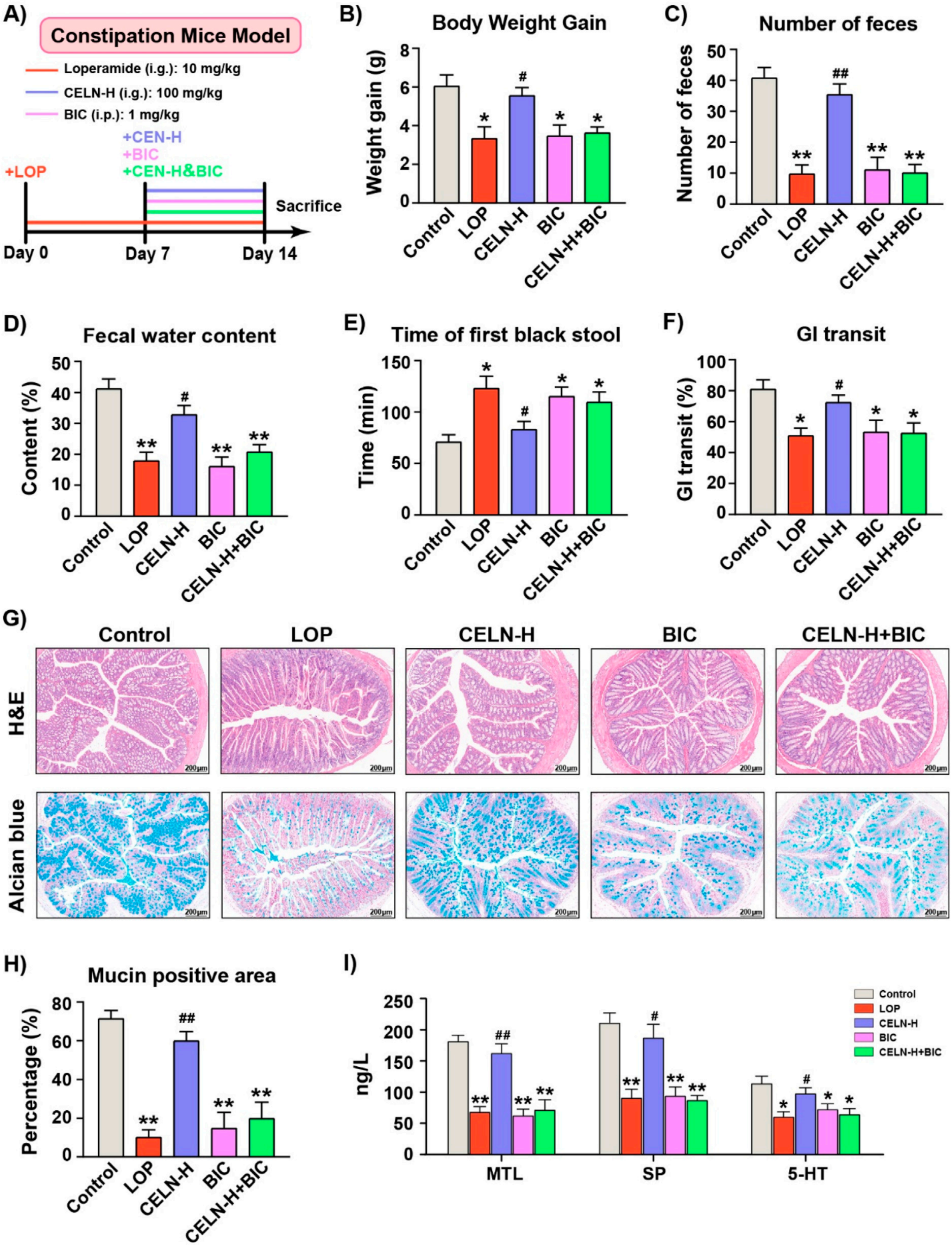
The effects of the CELNs depend on GABA_A_ R signaling pathway activation. **(A)** Animal experimental flowchart. **(B)** Body weight gain. **(C)** Number of fecal pellets. **(D)** Fecal water content. **(E)** Time to first black feces. **(F)** Gastrointestinal transit rate. **(G)** Representative images of H&E and alcian blue stained colon tissues from each group (scale bar = 200 µm). **(H)** The percentage of mucin-positive area (blue staining). **(I)** Serum concentrations of MTL, SP and 5-HT among the experimental groups. The data are presented as the means ± SEMs. ^*^ *P* < 0.05, ^**^ *P* < 0.01 vs. the Control group; ^#^ *P* < 0.05, ^##^ *P* < 0.01 vs. the LOP group.

To confirm the critical role of GABA_A_ R in the CELNs -mediated amelioration of constipation, mice with LOP-induced constipation were administered a high dose of CELNs by gavage (CELN-H group), intraperitoneally injected bicuculline (BIC group), or orally gavaged with a high dose of CELNs with concomitant intraperitoneal injection of bicuculline (CELN-H + BIC group), as illustrated in Figure 4A. Consistent with the previous results, CELNs treatment significantly attenuated constipation-associated parameters in mice, including body weight loss, fewer fecal pellets, decreased fecal water content, prolonged time to first black stool excretion, and slowed intestinal transit. However, administration of a GABA_A_ R inhibitor (bicuculline) abolished the therapeutic effects of CELNs, with no further improvement observed in these parameters (Figures 4B–F). Histomorphologically, CELNs treatment increased the thickness of the colonic muscle layer and the number of goblet cells in constipated mice. However, GABA_A_ R inhibitor administration abolished the effects of the CELNs (Figures 4G,H). Similarly, CELNs significantly increased the serum levels of GI motility-related hormones and neurotransmitters in constipated mice, but no such improvements were observed in the BIC and CELN-H + BIC groups (Figure 4I).

### 3.5 CELNs fail to improve constipation in mice treated with a GABA_A_ R inhibitor

**FIGURE 5:**
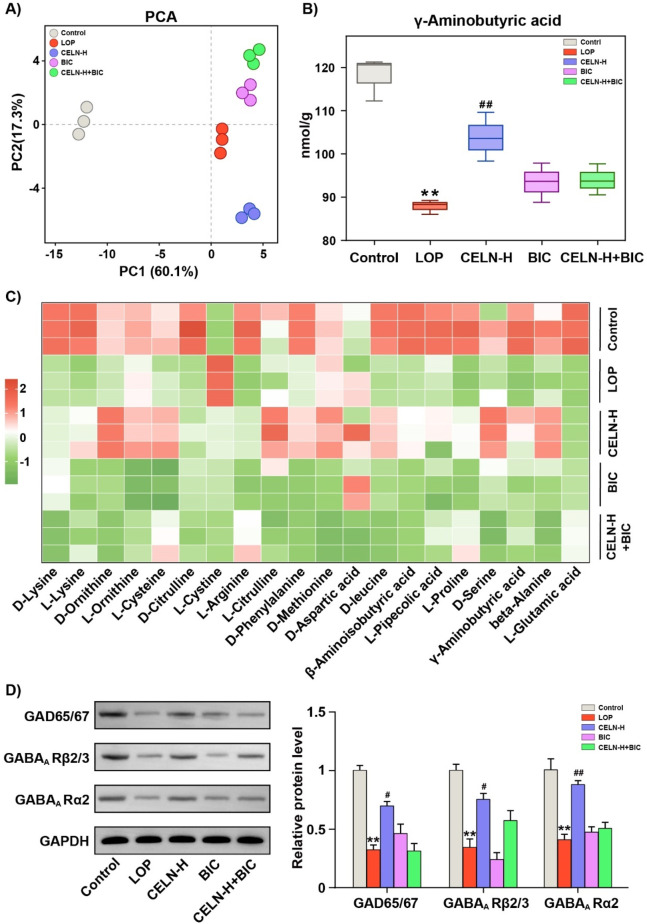
CELNs fail to improve constipation in mice treated with a GABA_A_ R inhibitor. **(A)** PCA of the targeted amino acid metabolomic data of the colonic contents of the mice in the five experimental groups. **(B)** GABA content in the colonic contents of each group. **(C)** Heatmap visualizing the differences in the levels of 20 amino acids among the five groups. **(D)** Western blot analysis of the expression of GAD65/67, GABA_A_ Rβ2/3 and GABA_A_ Rα2 in the colon; GAPDH was used for normalization. The data are presented as the means ± SEMs. ^**^ *P* < 0.01 vs. the Control group; ^#^ *P* < 0.05, ^##^ *P* < 0.01 vs. the LOP group.

To further explore the crucial role of GABA in the regulation of constipation by CELNs, we employed amino acid-targeted metabolomics. In all, 71 amino acids converged in the PCA plot. Figure 5A demonstrates pronounced cluster segregation across experimental cohorts. Consistent with previous results, CELNs significantly restored GABA levels in the colonic contents of constipated mice, but the GABA_A_ R antagonist completely blocked this therapeutic effect (Figure 5B). We subsequently selected 20 amino acids with relatively high contents, including L-glutamic acid and GABA, for heatmap analysis (Figure 5C). Western blot analysis demonstrated significantly reduced protein expression of GAD65/67, GABA_A_ R α2, and GABA_A_ R β2/3 in the LOP group compared to Controls, and CELNs intervention increased these protein expression levels. However, under bicuculline blockade, the effects of the CELNs were inhibited (Figure 5D).

### 3.6 CELNs ameliorate dysbiosis of the gut microbiota in constipated mice

**FIGURE 6:**
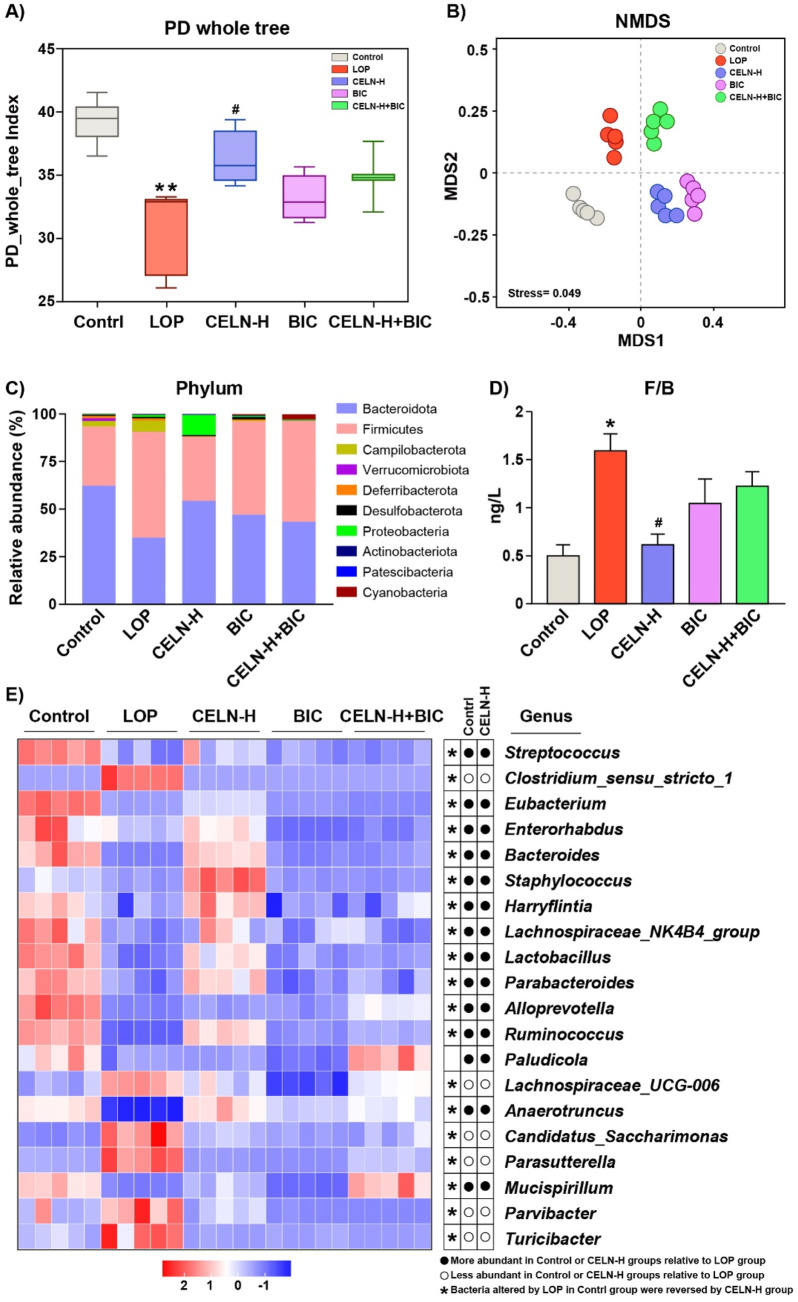
CELNs ameliorate gut microbiota dysbiosis in constipated mice. **(A)** Microbial richness indicated by Faith’s Phylogenetic Diversity (PD) whole tree. **(B)** Beta diversity assayed by non-metric multidimensional scaling (NMDS). **(C)** Changes in the gut microbiota at the phylum level. **(D)** The ratio of Firmicutes/Bacteroidetes. **(E)** Heatmap comparing the experimental groups at the genus level. The data are presented as the means ± SEMs. ^*^ *P* < 0.05 vs. the Control group; ^#^ *P* < 0.05 vs. the LOP group.

Given the established role of gut microbiota dysbiosis in constipation pathogenesis, we performed 16S rDNA sequencing to characterize fecal microbial diversity, richness, and community structure across experimental cohorts. α-Diversity metrics (calculated via PD Whole Tree) were significantly reduced in the LOP group versus controls (*P* < 0.01). CELN administration restored α-diversity to near-baseline levels (*P* < 0.05, vs. the LOP group). Analogously, α-diversity in BIC- and CELN + BIC-treated cohorts showed no significant deviation from the LOP group (Figure 6A). β-diversity analysis via NMDS revealed pronounced microbiota segregation among all five experimental groups, demonstrating distinct treatment-specific modulation of gut microbial ecosystems (Figure 6B). At the phylum level, Bacteroidetes and Firmicutes predominated across all cohorts, representing phylogenetically conserved taxa implicated in gastrointestinal pathophysiology. LOP-treated mice exhibited a dysbiotic signature characterized by depleted Bacteroidetes and enriched Firmicutes abundance (Figure 6C). This phylogenetic imbalance was significantly ameliorated following CELN intervention, demonstrating restoration of commensal microbial equilibrium. Phylum-level microbiota analysis further demonstrated that CELN intervention restored the Firmicutes/Bacteroidetes (F/B) ratio in the constipation model to levels statistically indistinguishable from baseline parameters (*P* > 0.05 vs. Control; Figure 6D). Furthermore, heatmap analysis was performed to explore how CELNs modulate the gut microbiota composition at the genus level. The abundance of 20 genera significantly differed among the experimental groups (Figure 6E). The results indicated significant changes in the abundance of most bacteria in the LOP group, with recovery of most of them to some extent after CELNs treatment. However, compared with those in the LOP group, there seemed to be no significant changes in the BIC and CELN + BIC groups at the genus level, except for a few bacteria.

### 3.7 Correlation and interaction network analyses of bacterial abundance and metabolic biomarkers

**FIGURE 7:**
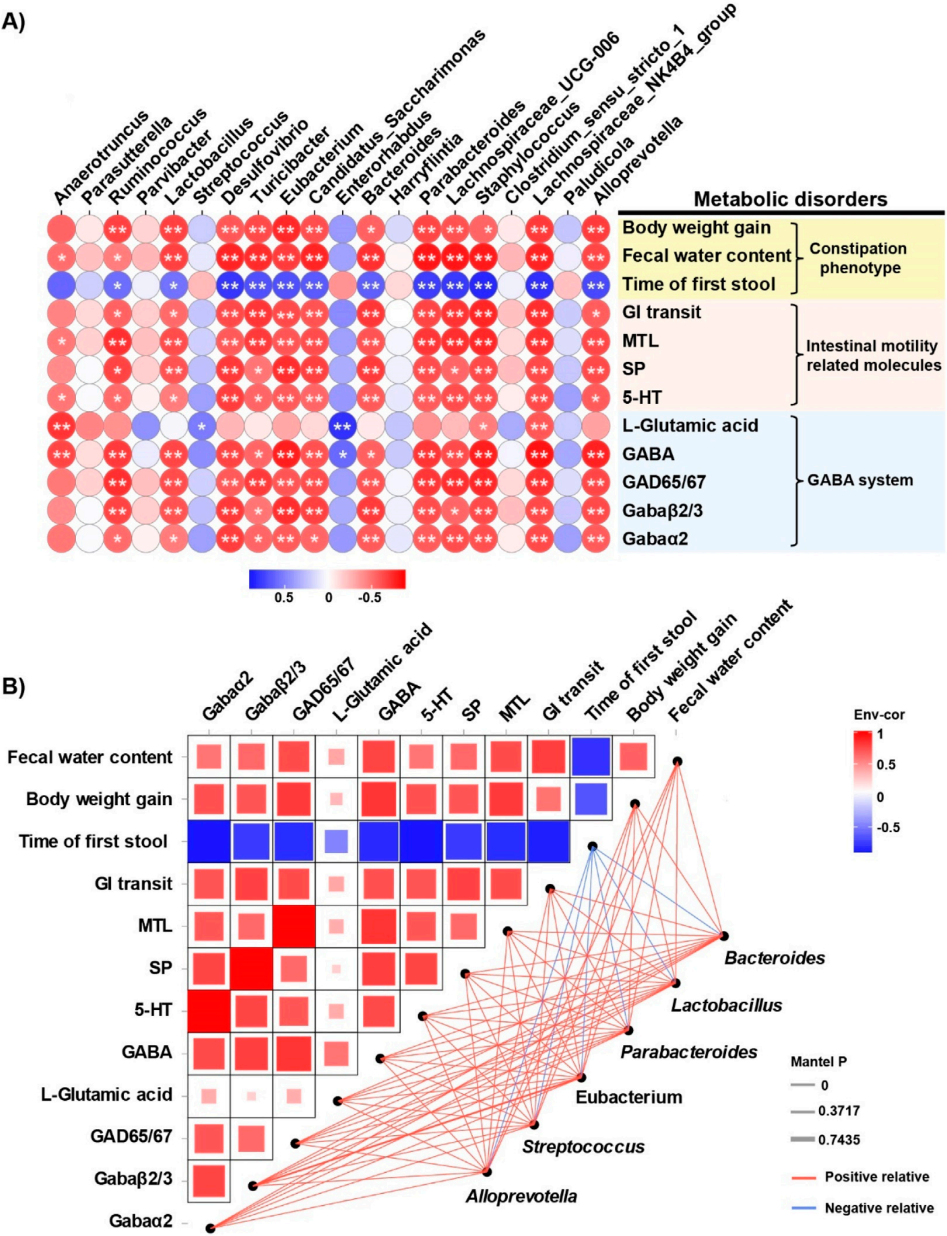
Correlation and interaction network analyses of bacterial abundance and metabolic biomarkers. **(A)** Spearman’s correlation analysis of intestinal bacterial abundance and constipation-related biomarkers. The colors range from blue (negative correlation) to red (positive correlation), and significant correlations are marked by ^*^ *P* < 0.05 or ^**^ *P* < 0.01. **(B)** Network heatmap of correlations among constipation-related biomarkers, GABA and its receptor and bacterial communities.

Spearman’s correlation analysis was performed to assess associations between constipation-related physiochemical parameters and gut microbiota shifts at the genus level (Figure 7A). Significant correlations were observed between constipation-associated physiological parameters, GABAergic signaling components and altered abundances of key genera including *Bacteroides*, *Lactobacillus*, *Parabacteroides*, *Eubacterium*, *Streptococcus* and *Alloprevotella*, etc. To further clarify the correlations among these bacteria, constipation phenotypes, and GABA and its receptors, we conducted an interactive network analysis (Figure 7B). The results revealed that GABA and its receptor system are positively correlated with intestinal motility, neurotransmitters and intestinal hormones, whereas bacteria such as *Bacteroides* are positively or negatively correlated with these factors to varying degrees.

## 4 Discussion

Constipation is the most common intestinal dysfunction condition worldwide. Large-scale epidemiological studies have demonstrated that there were statistically positive associations of constipation with breast and colon cancer, cardiovascular disease, risks of all-cause mortality, and cardiovascular disease mortality (Peng et al., 2022). Owing to the lack of diagnostic and treatment norms and the abuse of laxatives, constipation has continued to be a gastroenterological public health problem. *C. deserticola*, a traditional medicinal and edible plant, has demonstrated unique therapeutic advantages in constipation management in clinical practice (Liu X. et al., 2025). Studies have shown that the bioactive phenylethanoid glycosides of this herb alleviate constipation symptoms through multitargeted regulatory mechanisms, such as modulating intestinal transit rates, improving enteric neuron–glia network function, and restoring mucosal barrier integrity (Yin et al., 2024; Wang et al., 2024). Compared with conventional plant extracts, plant-derived exosome-like nanovesicles represent a novel natural nanoscale drug delivery system with superior biomembrane penetration efficiency (Kim et al., 2022; Zhao et al., 2024). In this study, we demonstrated that CELNs ameliorate LOP-induced constipation in mice by regulating GI hormones, the gut microbiota composition, and intestinal metabolites. Our findings highlight the potential of CELNs as a safe and effective nanotherapeutic strategy for constipation management.

In this study, we isolated and characterized CELNs, which exhibited a nanoscale particle size and negative zeta potential, and further validated their *in vitro* biological functionalities (Figure 1). We subsequently systematically investigated the therapeutic potential of CELNs in constipation management, providing multifaceted evidence for their efficacy. A LOP-induced murine constipation model in which LOP primarily activates intestinal μ opioid receptors, suppresses smooth muscle motility, and inhibits water absorption, thereby inducing constipation, was established (Camilleri, 2011). The results demonstrated that CELNs effectively counteracted LOP-induced constipation phenotypes, including by increasing fecal output and water content, shortening defecation latency, accelerating intestinal transit, and restoring colonic histology and goblet cell secretion, the last of which facilitates fecal excretion. Moreover, CELNs significantly elevated the serum levels of excitatory neurotransmitters (including SP and 5-HT) and gut hormones (including MTL) in constipated mice (Figure 2). MTL reportedly enhances GI smooth muscle motility and promotes peristalsis (Zhang et al., 2024), whereas SP strengthens intestinal contraction by activating sensory and motor neurons, thereby propelling luminal contents (Wu et al., 2025a). Additionally, 5-HT fine-tunes intestinal peristaltic rhythms via the enteric nervous system (ENS)–interstitial cell of Cajal (ICC) network (Liu K. et al., 2025). These findings collectively suggest that CELNs may serve as promising therapeutic agents for constipation by modulating gut neurotransmitters and hormones.

To elucidate the mechanisms underlying CELNs-mediated constipation alleviation, we employed host colonic transcriptomics to elucidate their molecular regulatory pathways. Comparative analysis of gene expression profiles between the LOP and CELN groups unexpectedly revealed a central role for GABA-related signaling. Specifically, CELNs treatment significantly upregulated GABA_A_ R subunits (including α3 and β2) and GABA synthases (including GAD1) in colonic tissues (Figure 3), suggesting that GABA and its receptor system play pivotal roles in CELNs-induced constipation relief. This finding aligns with those of prior studies demonstrating that intestinal GABA_A_ R is highly sensitive to GABA and sustains tonic activation, which facilitates rhythmic smooth muscle contraction (Masiulis et al., 2019).

GABA is a major neurotransmitter in the body that regulates GI function by influencing the ENS and plays a significant role in regulating GI motility by modulating intestinal fluid and electrolyte transport (Gros et al., 2021). Consistent with these findings, our results demonstrated that the significant therapeutic effect of CELNs was completely abolished after the administration of a GABA_A_ R antagonist (bicuculline), and no further improvement in the constipation phenotype was observed (Figure 4). These findings further confirmed that the GABA_A_ R-mediated signaling pathway is the core mechanism by which CELNs alleviate constipation. Additionally, studies have shown that GABA stimulates the release of neurotransmitters such as acetylcholine in intestinal neurons by activating GABA_A_ R, directly affecting the contraction of intestinal smooth muscle (Seifi et al., 2014). In our study, we also found that CELNs significantly restored GABA levels in the feces of constipated mice, but the GABA_A_ R antagonist completely blocked this therapeutic effect, indicating that this action depends on the activation of the GABA_A_ R signaling pathway (Figure 5B).

A recent study further demonstrated that in the intestine, GABA_A_ R activation is usually associated with depolarization, causing excitation of intestinal neurons (Gajic Bojic et al., 2025). Interestingly, GABA_A_ R in the intestine are composed mainly of α, β, and γ subunits, and GABA_A_ R with different subunit compositions have different regulatory effects on intestinal smooth muscle contraction (Auteri et al., 2014). For example, the activation of GABA_A_ Rs with α1-γ2 subunits can increase the contractility of intestinal smooth muscle, whereas the activation of receptors with α4-5 subunits may have the opposite effect (Gajic Bojic et al., 2025; Alexopoulos and Dalakas, 2019). The α2, α3, and γ2 subunits are mainly expressed in the human colon, with the α2 subunit being the most abundant. Expression of the β2/3, π, and δ subunits has also been reported in animal models. Our results indicate that CELNs can increase the protein expression levels of GAD65/67, GABA_A_ Rα2, and GABA_A_ Rβ2/3 but that this effect depends on the integrity of the GABA _A_ R signaling pathway (Figures 5D,E). This result is also supported by previous studies, in which colonic epithelial GAD67 inhibited the release of the proinflammatory factor TNF-α and alleviated inflammation-mediated intestinal motility disorders (Kinoshita et al., 2006; Younis et al., 2024). GABA enhances the activity of the anoctamin 1 chloride channel through the α2 receptor subunit, thereby increasing the propagation efficiency of colonic ICC (Munoz et al., 2020). The β2/3 subunits of GABA_A_ R are also distributed on intestinal neurons, and their activation can regulate the postsynaptic potential to affect neuronal excitability and, in turn, regulate intestinal smooth muscle contraction (Auteri et al., 2015a).

Intestinal motility is regulated by the interactions among the intestinal microbiota, microbial-derived products, and the ENS (Marques de Souza et al., 2025). An imbalance in the intestinal microbiota can lead to reduced intestinal secretion, impaired colonic epithelial integrity, and slowed intestinal peristalsis (Bai et al., 2022). For example, tea flower-derived polysaccharides alleviate dysbiosis by normalizing the F/B ratio, inhibiting pathogenic genera, and promoting beneficial genera (Wu et al., 2025b). Garlic polysaccharides reduce the relative abundance of harmful bacteria and the F/B ratio, restore intestinal homeostasis, and alleviate LOP-induced constipation symptoms (Xie et al., 2024). Consistent with other studies on constipation interventions, our results revealed that CELNs intervention improved the reduction in species richness and diversity caused by LOP. A recent study by Yu et al. demonstrated that *C*. *polysaccharides* alleviated constipation by modulating the gut microbiota and restoring intestinal barrier function (Yu et al., 2024). Similarly, we found that CELNs reshaped the disrupted microbiota structure, decreasing the F/B ratio and promoting beneficial genera, underscoring a common therapeutic pathway shared by different bioactive components from the same herb (Figures 6A–D). At the genus level, CELNs increased the relative abundances of beneficial bacteria such as *Lactobacillus*, *Bacteroides*, *Streptococcus*, *Parabacteroides* and *Eubacterium* (Figure 6E). Notably, many species of *Lactobacillus* can promote the production of SCFAs such as acetic acid, lower the intestinal pH, and thereby stimulate intestinal smooth muscle contractions. *Streptococcus* can synergize with *Lactobacillus* to further enhance this effect (Fang et al., 2025). In patients with functional constipation, *Bacteroides* is present in lower amounts, and its upregulation is associated with improved constipation. *Parabacteroides* can produce secondary bile acids (such as ursodeoxycholic acid), repair intestinal wall integrity, and enhance intestinal function. *Eubacterium* mainly produces butyric acid to regulate intestinal motility. On the other hand, after CELNs treatment, the abundances of other harmful bacteria related to constipation, such as *Clostridium_sensu_stricto_1* and *Parasutterella*, have been found to be significantly reduced (Gao et al., 2024).

The interaction between the gut microbiota and the host is achieved through complex pathways mediated by multiple signaling molecules. In recent years, multiomic approaches (integrating the gut metagenome, metabolome and host transcriptome) have been used to systematically analyze the associations among microbial metabolites and host physiological mechanisms. On the basis of spearman correlation and interaction network analyses, we investigated the crosstalk among the gut microbiota, amino acid metabolites and constipation-related biomarkers. The results revealed that beneficial bacteria such as *Lactobacillus*, *Bacteroides*, *Streptococcus*, *Parabacteroides* and *Eubacterium* were strongly positively correlated with GABA and its receptor system (Figure 7A). Critically, our findings demonstrate that CELNs intervention robustly increased the abundance of these specific GABA-producing genera, thereby providing a direct microbial basis for the elevated colonic GABA levels. While our 16S rDNA sequencing identified enrichment at the genus level (e.g., *Bacteroides*), a recent mechanistic study by Xia et al. (Xia et al., 2024) provides crucial species-level insight. They demonstrated that oral administration of *Lacticaseibacillus rhamnosus* LRJ-1 alleviated constipation in mice accompanied by increasing fecal GABA levels and intestinal commensal *Bacteroides*, and GABAergic synapses activation. Furthermore, administration of either *B. uniformis* ATCC 8492 or GABA alleviated constipation and increased gastrointestinal motility in constipated mice. Although our current data cannot confirm the specific enrichment of *B. uniformis*, the significant increase in the *Bacteroides* genus we observed aligns with this paradigm. We therefore propose a plausible mechanism whereby CELNs may function as prebiotic-like nanomodulators, creating a gut microenvironment that favors the proliferation of GABA-producing bacteria, potentially including key species such as *B. uniformis*. This microbial shift would be expected to enhance the expression of bacterial GAD, thereby elevating luminal GABA levels (Strandwitz et al., 2019; Casertano et al., 2024; Park et al., 2005; Han et al., 2020). The subsequent activation of host GABA_A_ receptor signaling on intestinal neurons and smooth muscle cells represents a convergent pathway to potentiate intestinal peristalsis (Hyland and Cryan, 2010; Greenwood-Van Meerveld et al., 2017; Auteri et al., 2015b). This finding is also supported by the results of the interaction network analysis (Figure 7B), which revealed that the levels of GABA and its receptor system are positively correlated with intestinal motility, neurotransmitter levels and intestinal hormones, suggesting that GABA stimulates​ intestinal peristalsis through multiple convergent pathways. This proposed mechanism aligns our findings with the established paradigm of microbiota-metabolite-host axis regulation.

It is noteworthy that our study was conducted in young adult male mice to first establish a clear proof-of-concept under controlled conditions. Given the higher prevalence of constipation in elderly and female populations, future studies employing aged models and considering sex as a biological variable will be crucial to fully evaluate the translational potential of CELNs.

## 5 Conclusion

In summary, we demonstrated that CELNs derived from *C. deserticola* have a strong regulatory effect on LOP-induced constipation in mice. Specifically, CELNs alleviate constipation by remodeling the gut microbiota, particularly by increasing the abundance of GABA-producing genera such as *Lactobacillus* and *Bacteroides*, thereby increasing intestinal GABA bioavailability and specifically activating GABA_A_ R subunits (e.g., α2 and β2/3) in a GABA_A_ R-dependent manner to potentiate intestinal peristalsis. Additionally, this study systematically elucidated the molecular mechanism by which the intestinal microbiota and its metabolite GABA improve constipation through activation of the GABA_A_ R signaling axis, providing a theoretical foundation for the development of constipation treatment strategies that target the interactions among the gut microbiota and its metabolites.

## Data Availability

The datasets presented in this study can be found in online repositories. The names of the repository/repositories and accession number(s) can be found in the article/Supplementary Material.

## Abbreviations

CELNs, Cistanche deserticola-derived exosome-like nanoparticles; GABA, γ-aminobutyric acid; GABAA R, GABA-A receptor; LOP, Loperamide; SCFAs, Short-chain fatty acids; GAD 65/67, Glutamic acid decarboxylase 65/67; TEM, Transmission Electron Microscopy; NTA, Nanoparticle tracking analysis; MTL, Motilin; SP, Substance P; 5-HT, 5-hydroxytryptamine; LC-MS, Liquid chromatography-tandem mass spectrometry; PCA, Principal component analysis; OTUs, Operational taxonomic units; NMDS, Non-metric dimensional scoring; ENS, Enteric nervous system; ICC, Interstitial cell of Cajal.
