# Supplementary material for: Cistanche deserticola-derived exosome-like nanovesicles target the Microbiota-GABA signaling axis to ameliorate loperamide-induced constipation
Source: Front Pharmacol. 2025 Nov 7;16:1693366. doi: 10.3389/fphar.2025.1693366 (PMC12634381; doi:10.3389/fphar.2025.1693366)
Supplement: Supplementary file 1 [file Supplementaryfile1.docx]

**Supplementary information**

**Supplementary methods**

1. Composition Analysis of CELNs.

**Supplementary figures**

Supplementary Fig.1. Composition Analysis of CELNs.

Supplementary Fig.2. Relative expression level of mucin1.

**Supplementary methods**

**1. Composition Analysis of CELNs**

High-performance liquid chromatography (HPLC) analysis was conducted using an Ultimate 3000/Agilent 1260II chromatography system). Chromatographic separation was achieved on an XY08 column using an isocratic mobile phase of methanol and 0.1% aqueous formic acid at a flow rate of 1.0 mL/min. The column temperature was maintained at 30 °C, and detection was performed at 330 nm. Sample solutions were prepared by ultrasonication in methanol for 30 min, filtered through a 0.22 μm membrane, and diluted 2-fold. These sample solutions, along with reference standards of echinacoside (91.8% purity) and verbascoside (97.6% purity), were injected (10 μL) under identical chromatographic conditions. The contents of the two phenylethanoid glycosides in Cistanche deserticola exosome-like nanovesicles (CELNs) were quantified using the external standard method based on peak area. System suitability criteria required a theoretical plate count (N) ≥ 5000 for echinacoside, a resolution > 1.5 between critical pairs, and a relative standard deviation (RSD) ≤ 2% for five consecutive injections of the reference standard solution.

**Supplementary figures**


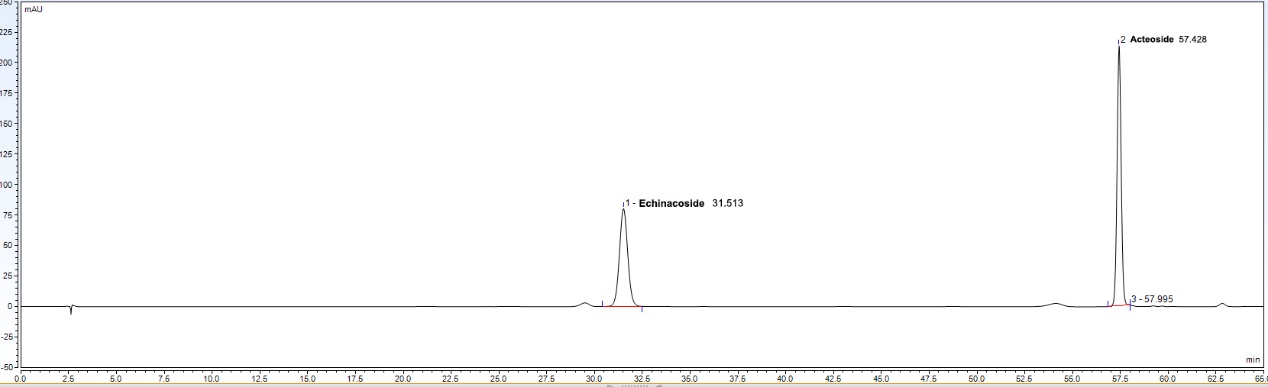


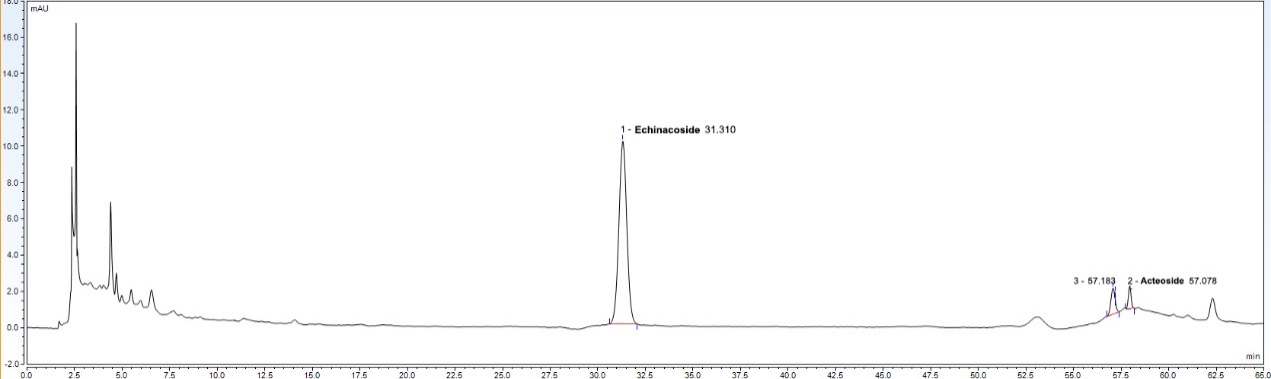


**Supplementary Fig.1.** Composition Analysis of CELNs.

**Supplementary Fig.2.** Relative expression level of mucin1. ^**^*P* < 0.01 *vs.* the CELN-H group.
