# Supplementary figures and images for: Cistanche deserticola-derived exosome-like nanovesicles target the Microbiota-GABA signaling axis to ameliorate loperamide-induced constipation
Source: Front Pharmacol. 2025 Nov 7;16:1693366. doi: 10.3389/fphar.2025.1693366 (PMC12634381; doi:10.3389/fphar.2025.1693366)

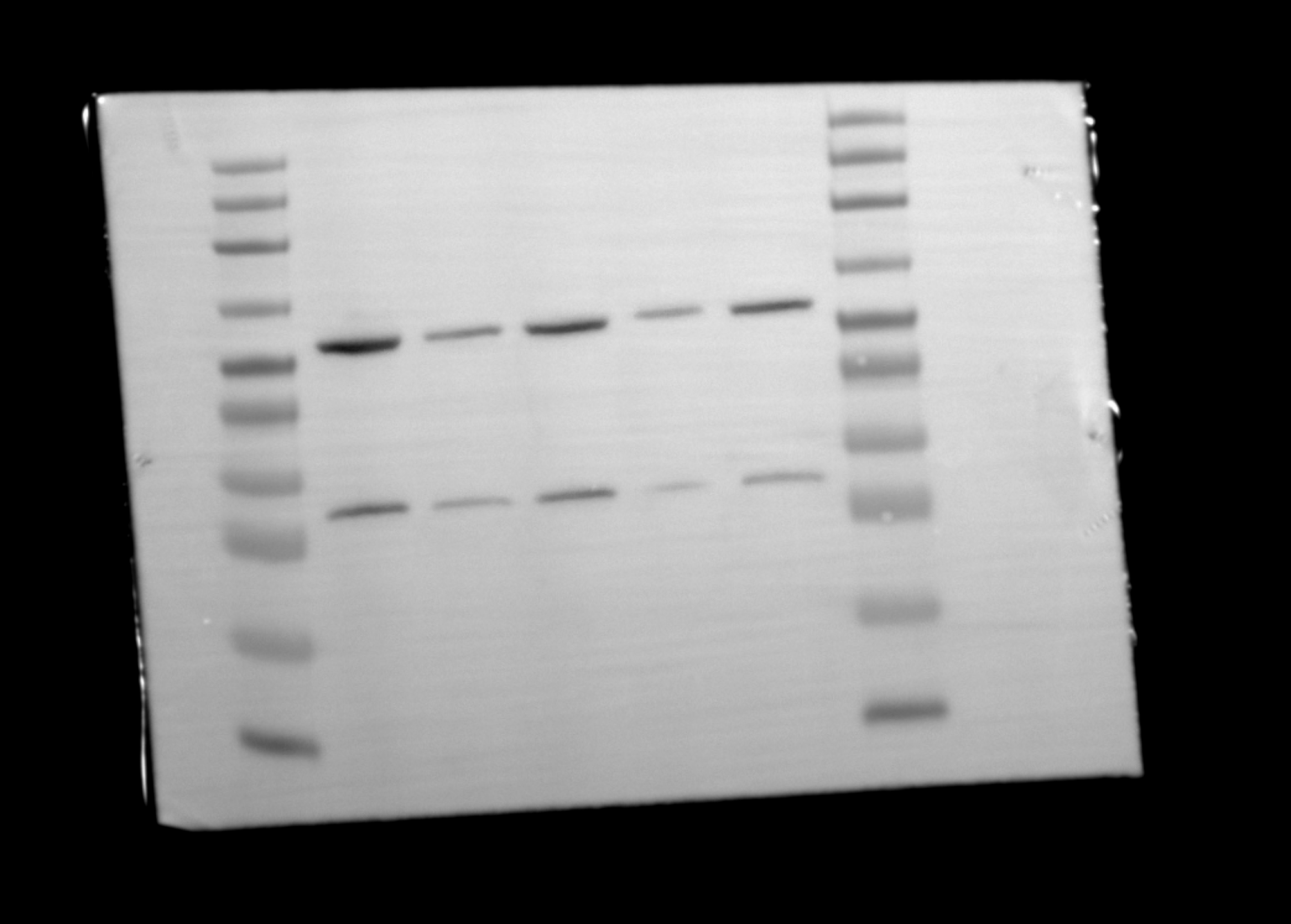

Supplement: Supplementary file 2 [file Supplementaryfile3.zip › Original Images for Blots/GABAA R a┬ 2 3.tiff]

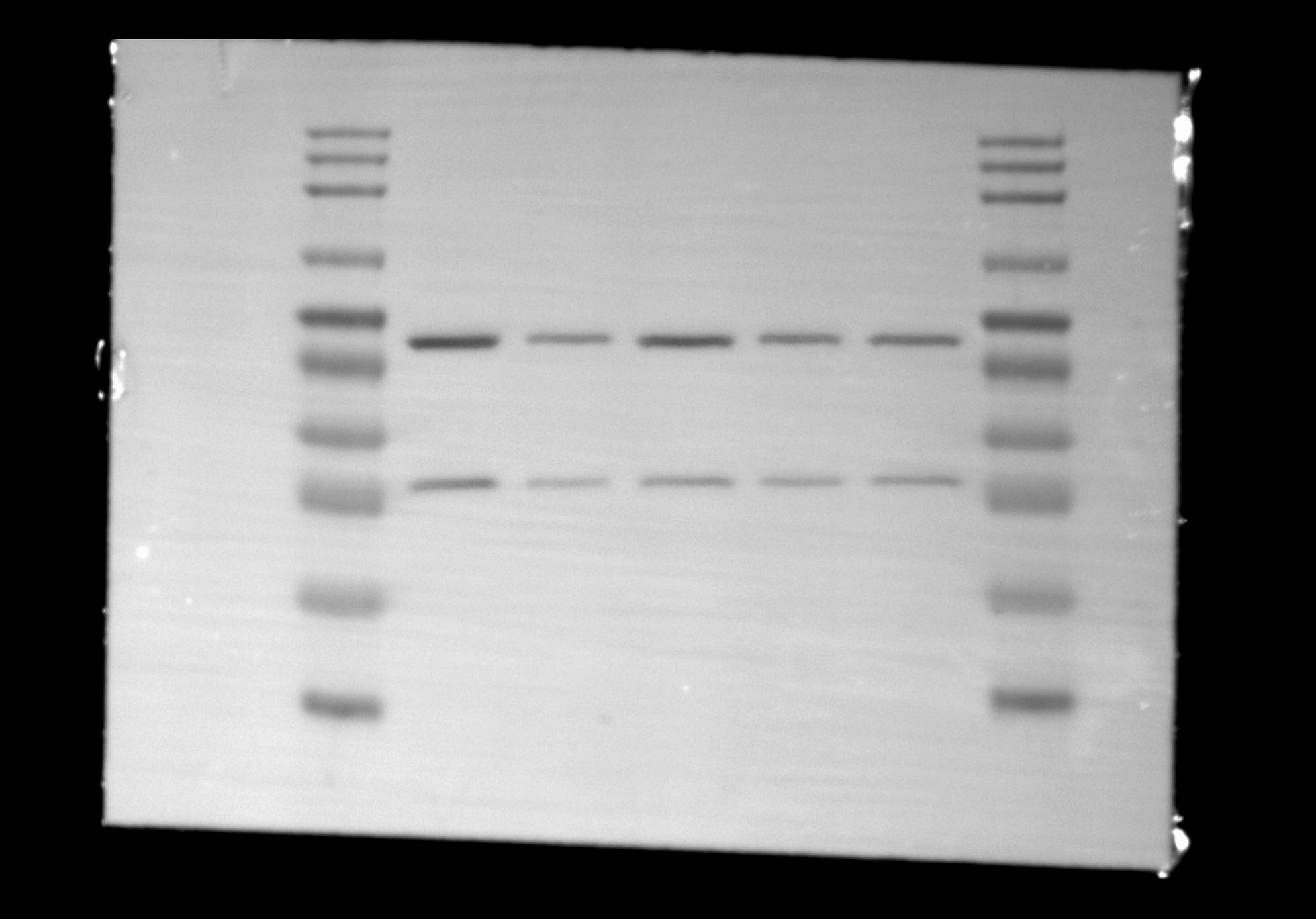

Supplement: Supplementary file 2 [file Supplementaryfile3.zip › Original Images for Blots/GABAA Ra┴2.tiff]

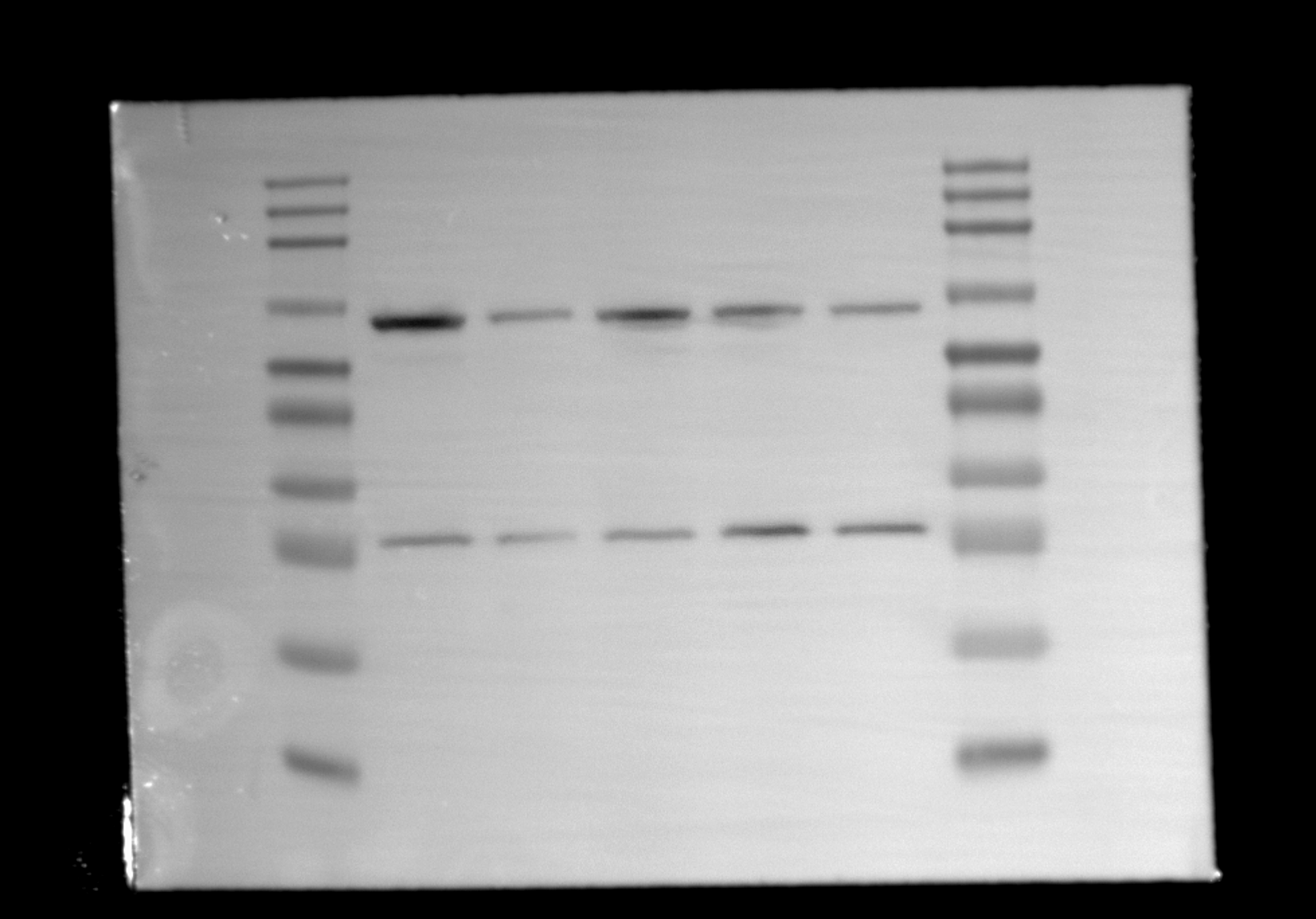

Supplement: Supplementary file 2 [file Supplementaryfile3.zip › Original Images for Blots/GAD65 67.tiff]

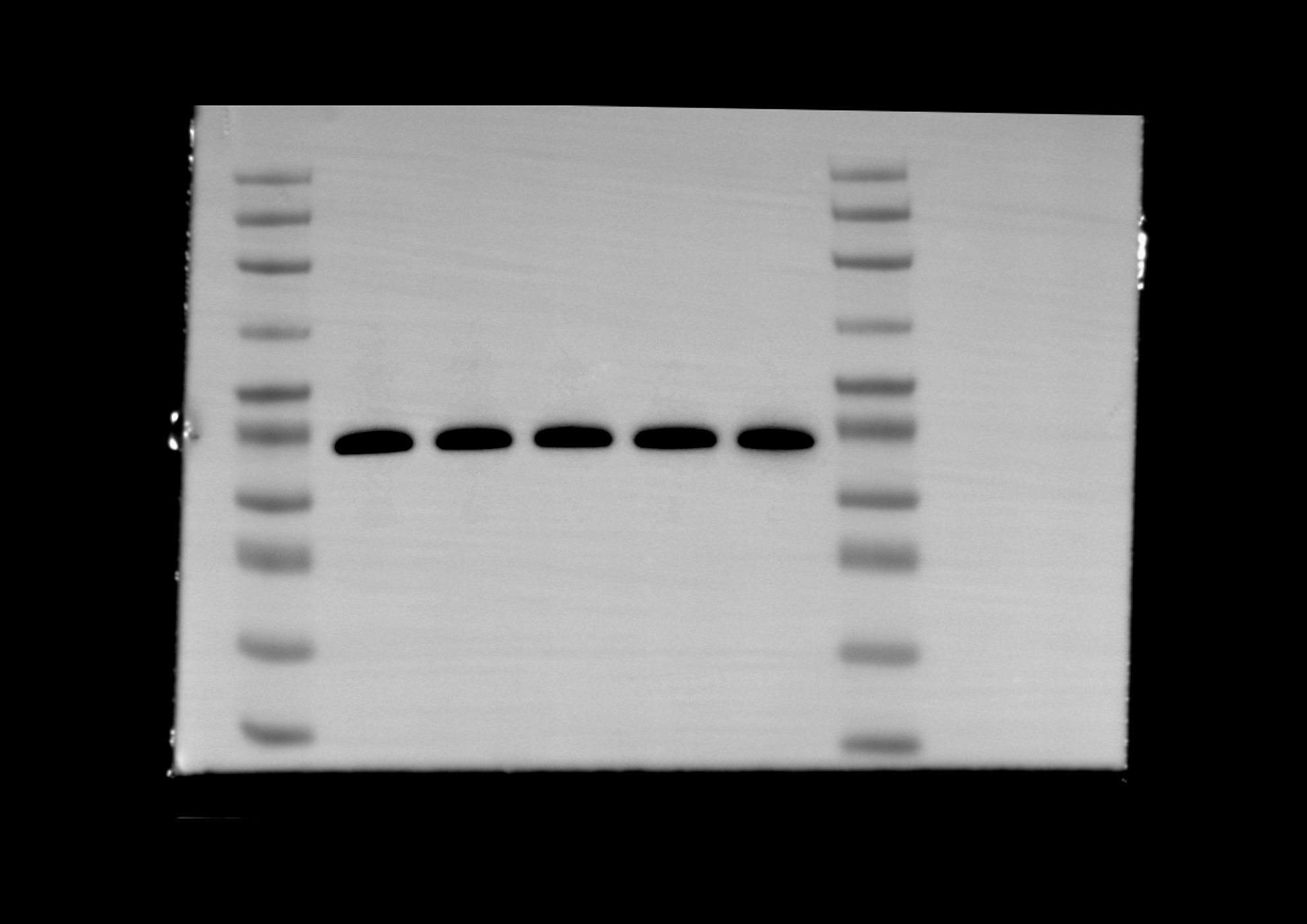

Supplement: Supplementary file 2 [file Supplementaryfile3.zip › Original Images for Blots/GAPDH.tiff]
